# Male‐Male Greeting Behavior Observed in Chacma Baboons ( *Papio ursinus griseipes* ) in Gorongosa National Park, Mozambique

**DOI:** 10.1002/ajpa.70169

**Published:** 2025-12-26

**Authors:** Jana Muschinski

**Affiliations:** ^1^ Primate Models for Behavioural Evolution Lab, School of Anthropology and Museum Ethnography University of Oxford Oxford UK

**Keywords:** baboons, cooperation, male greeting, notification, *Papio* sp.

## Abstract

**Objectives:**

Male–male greetings have been described across many primate species, with varying forms and functions. Within *Papio,* their study has been of particular interest as baboons show variation in greeting, male–male cooperation, philopatry, and social systems. The function of greetings in *Papio* may differ by species, including facilitating cooperative behavior, negotiating rank, and maintaining social relationships. Chacma baboons, a species that generally exhibits limited to no coalition formation and low male–male tolerance, remain understudied regarding their male–male greeting behavior.

**Materials and Methods:**

Here I present descriptions of male–male greeting behavior of chacma baboons ( *Papio ursinus griseipes* ) recorded in Gorongosa National Park, Mozambique in 2018 and 2019. Behavioral data was collected from video footage and categorized using established definitions from the published literature (e.g., presence and intensity of physical contact, reciprocity, and completeness).

**Results and Discussion:**

Strong similarities in signal use between the sample of chacma baboon greetings and published accounts of olive, hamadryas, and yellow baboon greetings were identified. Specifically, rates of physical contact, intense physical contact, and reciprocal use of facial expressions were similar to those of the other *Papio* species, excluding the Guinea baboon which exhibits more stereotyped and highly physical greetings. The proportion of observed chacma baboon greetings which were considered “incomplete” (i.e., exhibiting only facial signals, with no presenting or contact) however, was greater than in the other baboon species, highlighting a key difference that may reflect the lower level of male tolerance and weaker male relationships in chacma baboons compared to other baboon species.

## 1 Introduction

Non‐aggressive, ritualized interactions, often involving brief physical touch, have been identified across a number of primate species and are often referred to as “greetings” (Corewyn and Setchell 2019; Dias and Rangel‐Negrín 2017; De Marco 2017). While greetings are seen in interactions between various sex‐combinations depending upon the species (e.g., female greetings in black‐and‐white colobus monkeys—Kutsukake et al. 2006), greetings between males are of particular interest because they often involve high‐risk physical contact such as genital touching (De Marco 2017). The functions of male greetings may differ by species, population density, and even individual or dyad (Corewyn and Setchell 2019). Conflict management, tension reduction, social bonding, confirming or signaling of rank, and coordinating action have been suggested as functional explanations of male greeting behavior across different species (Corewyn and Setchell 2019; Mercier et al. 2017; De Marco 2017).

Male greeting behavior has been particularly well‐studied in baboons (Dal Pesco and Fischer 2020), at least partly because the high level of variation in social structure and organization in the genus provides an ideal setting to study greeting behavioral diversity and its relationship with philopatry and aspects of male–male relationships such as spatial tolerance and cooperation. Six species of baboon currently range through a variety of environments across Africa and the Arabian Peninsula ( *Papio hamadryas* —hamadryas baboon, *P. papio*—Guinea baboon, *P. anubis*—olive baboon, *P. cynocephalus*—yellow baboon, *P. ursinus—*chacma baboon, *P. kindae—*kinda baboon), with several hybridisation zones (Roos et al. 2021; Zinner et al. 2013).

The four “COKY” baboons (chacma, olive, Kinda, and yellow baboons; Jolly 2020) all exhibit multi‐male, multi‐female groups with polygynandrous mating systems, female philopatry, and male dispersal (Henzi and Barrett 2003). These species differ considerably in their coalitionary behavior, with male–male coalitions having been reported consistently in olive and yellow baboons, but reported rarely in chacma baboons even after decades of study (Henzi and Barrett 2003, Henzi and Barrett 2005; but see Saayman 1971). Kinda baboons have only been studied relatively recently (Petersdorf et al. 2019; Weyher et al. 2014, 2025), with coalitionary behavior not yet reported.

Unlike the COKY baboons, Guinea and hamadryas baboons exhibit multi‐level hierarchical social structures. In both species, males are predominantly philopatric, usually remaining in their natal clan/party, while females disperse from their natal groups (Fischer et al. 2017; Kummer 1984). In hamadryas baboons, the basic social building block is a one‐male unit, consisting of a leader male, a variable number of females, and a follower male who is typically maternally related to and lower ranking than the leader male (Romero and Castellanos 2010; Schreier and Swedell 2009; Städele et al. 2016). These units come together to form clans and then bands (Schreier and Swedell 2009). Unlike hamadryas baboons, male Guinea baboons demonstrate strong bonds with other males, with high levels of male–male tolerance and affiliative behaviors such as grooming, even between distantly related males (Patzelt et al. 2014). Male Guinea baboons have dyadic association preferences which at times last several years, and these preferred association partners often also feature as coalition partners (Dal Pesco et al. 2021; Fischer et al. 2017). The strong male–male bonds seen in Guinea baboons are also reflected in their frequent and highly physical male greetings (Dal Pesco and Fischer 2018; Dal Pesco et al. 2021).

In baboons, “greeting” refers to approaches between males which involve some combination of a swaggering gait, ear‐flattening, lip‐smacking, presenting, and, depending upon species, physical contact behaviors including mounting, hip grasping, and genital touching (Colmenares 1990; Dal Pesco and Fischer 2018, 2020; Mondada and Meguerditchian 2022). Baboon species with higher rates of greetings involving physical elements also tend to exhibit higher degrees of spatial tolerance and increased male coalitionary behavior, as in the case of the Guinea baboon for example (Dal Pesco and Fischer 2020, 2018). Male greetings occur in hamadryas and Guinea baboons, and at a lesser rate, yellow and olive baboons (Hausfater and Takacs 1987; Smuts and Watanabe 1990; Dal Pesco and Fischer 2018; Fraser and Plowman 2007). The most physical and intense of greetings are exhibited by Guinea baboons; more varieties of physical contact are exhibited, and physical contact is generally more frequent and risky than in the other species (Dal Pesco and Fischer 2018, 2020; Whitham and Maestripieri 2003). Greetings take on a variety of functions in male baboons, and may assist with in‐group identification, bond testing, and relationship reinforcement (Guinea baboons—Dal Pesco and Fischer 2018; Whitham and Maestripieri 2003), indicate willingness to cooperate or be used to negotiate forming of coalitions (olive baboons—Smuts and Watanabe 1990; Watanabe and Smuts 2004), or ease tension and avoid confrontation through signaling of competitive power (hamadryas baboons—Colmenares 1990, Colmenares 1991a; Fraser and Plowman 2007). It has been suggested that in baboons, and other primates such as macaques, there is a link between communicative repertoire—including the presence and intensity of male greetings—and characteristics of the social structure, such as level of despotism, linearity of rank and intensity of male competition (Dal Pesco and Fischer 2020; Kavanagh et al. 2021; Whitham and Maestripieri 2003; Maestripieri 2005; De Marco et al. 2014). Within baboon greeting behavior in particular, increased tolerance and cooperation between males appears to be associated with higher rates of and more physical greeting behavior (Dal Pesco and Fischer 2020).

While greetings of other baboon species are relatively well studied, the study of greeting in chacma baboons and kinda baboons is limited. Chacma baboons are generally reported as exhibiting limited greeting behavior, with little to no physical or high‐risk (i.e., genital) contact (Henzi et al. 2008; Kalbitzer et al. 2015) and greeting behavior in chacmas has been referred to as “virtually absent” in the literature (Dal Pesco and Fischer 2018). Chacma baboons exhibit some of the less intense greeting behaviors reported in other species (Kalbitzer et al. 2015; Saayman 1971; Hall 1962). However, close proximity approaches by male chacma baboons, whether towards a recipient adult male or adult female, are unlikely to include contact greeting behaviors (Kalbitzer et al. 2015; Saayman 1971; Hall 1962). This may be due to the low levels of social tolerance and high rates of aggression between male chacma baboons (Dal Pesco and Fischer 2020; Kalbitzer et al. 2015). Given the limited research on chacma baboon greetings, further research would add to our understanding of behavioral diversity within *Papio* and how this relates to other species characteristics such as male tolerance, coalition formation, and competition.

Here I provide a further account of male greeting behavior in chacma baboons and situate this within the existing greeting literature. The video footage used in this study was recorded in 2018 and 2019 for use in other projects (Baehren and Carvalho 2022). During the COVID‐19 pandemic, when fieldwork was not possible, behavioral data was collected from the video footage for the purpose of studying male greeting behavior. The primary purpose of this work is to provide an in‐depth description of the observed set of greetings, supplementing the limited published data on male–male greeting in chacma baboons. Based on the existing literature, the Gorongosa chacma baboons were expected to exhibit less contact behavior, particularly intense contact, during greetings when compared to other baboon species. It is not uncommon for interactions between male baboons to lead to fights and injuries, with injury also contributing to overall mortality (Drews 1996; Taniguchi and Matsumoto‐Oda 2018), and we would therefore expect species with lower male spatial tolerance and higher male–male competition, such as chacma baboons, to be less likely to exhibit high‐risk greeting.

In this study, the total number of male–male greetings reported cannot be directly compared to hourly rates reported elsewhere due to differences in methodology (opportunistic videography vs. focal follows), so I will not discuss hourly rates of greeting. Testing of functional hypotheses was also not possible due to the small sample size, limited information on individual identity, and lack of rank data. Thus, the primary purpose of this work is to describe the form (i.e., behavioral elements, completeness, intensity, and reciprocity) of male greeting behavior within the Gorongosa chacma baboons, providing a point of comparison with the existing *Papio* literature. Where possible, comparisons of greetings of different age‐class combinations are presented to provide additional context and some insight into the ontogeny of greeting behavior.

## 2 Methods

### 2.1 Study Site and Population

Fieldwork was conducted in Gorongosa National Park, Mozambique, located in the southern end of the East African Rift System (Bobe et al. 2023; Macgregor 2015; Martinez et al. 2019; Tinley 1977). The park covers 3770 km^2^ and has a mosaic ecosystem with high biodiversity and recovering megafauna and carnivore populations (Stalmans et al. 2020, 2019; Correia et al. 2017). There are over 230 baboon troops ranging throughout the park (Stalmans et al. 2022; Beardmore‐Herd et al. 2025; Hammond et al. 2025; Biro et al. 2025). The baboons exhibit phenotypic traits seen in chacma, yellow, and kinda baboons (Martinez et al. 2019), with genetic data indicating they are best classified as chacma baboons ( *Papio ursinus griseipes* ) with evidence of past complex gene flow with yellow baboons, but no recent gene flow (Santander et al. 2022; da Ferreira Silva et al. 2025; Caldon et al. 2025). Gorongosa National Park is situated 100 km south of the suggested hybridisation zone between southern yellow baboons and northern chacma baboons (Martinez et al. 2019; Santander et al. 2022; da Ferreira Silva et al. 2025).

This study involved the collection of behavioral data from video footage of two troops, the Chitengo troop and the Montebelo troop, both of which range around the tourist lodge and research centre of the national park (Farassi et al. 2025). The Chitengo troop is highly habituated to the presence of humans and can be approached to within 5 m, while the Montebelo troop is less habituated, avoiding human approach within 15–20 m. The tourist and research centre consists of several intersecting roads, a restaurant area and main lodge, a ranger station, tourist and researcher cabins, a pool, a recreation area, and other infrastructure. The baboons typically forage in this area for several hours during the day, while spending the late afternoon through early morning in the surrounding savannah woodland. During the 2019 field season, the Chitengo troop had eight resident adult males, three peripheral adult males, one large juvenile male, 11 adult females, one subadult/large juvenile female, approximately 15 further juveniles, and an indeterminate number of infants (five births recorded between July and November 2019). The Montebelo troop had an estimated troop size of 40 to 50 individuals during 2019, with 14 adult females and 12 adult males identified as of 2019. The number of individuals in each age and sex class was unknown, but in addition to the aforementioned identified adult males and adult females, a minimum of three subadult males resided in the group at the time of filming. The author identified all Chitengo troop adults, subadults, and large juveniles during the 2019 field season, but the Montebelo troop remained largely unidentified at the time.

Filming of both troops was completed between October and November 2018 by Baehren (Baehren and Carvalho 2022) and in July through November 2019 by the author, amounting to a total of 65 h of footage. All filming was done opportunistically, with the camera centered on groups of baboons seated near each other, rotated throughout the day. Filming distance was variable due to environmental limitations and the radius captured around individuals ranged from three to approximately 20 m depending upon filming distance and location of the individual within the frame. Visibility restrictions were accounted for during video coding (see “Data Collection” below). Recording could not be randomized or focal follows completed because individuals were not identified at the time of filming. Recordings were completed across varying contexts while following the troop and spread throughout the day (i.e., between the hours of approximately 6:30 am and 4 pm). Fieldwork was conducted under permit number PNG/DSCi/C145/2019 (J. Muschinski) and PNG/DSCi/C110/2018 (L. Baehren) and was approved by the University of Oxford Animal Welfare and Ethical Review Board (APA/1/5/ACER/10Dec2018).

While other studies of baboon greeting behavior have not relied on video footage, the use of video footage to study communication and signaling in primates is common (e.g., Grund et al. 2023). This method makes it possible to rewatch footage to confirm coding of behaviors, making it less likely a coder will miss less salient signals. This can be especially useful when observing an interaction where multiple signals are co‐occurring or when having to record signals being exhibited by two individuals simultaneously.

### 2.2 Data Collection

I reviewed all video footage and identified instances of male greeting, defined in line with Smuts and Watanabe (1990). An interaction was considered a greeting when a male approached another male, typically accompanied by direct gaze, and performed at least one of the following behavioral elements: ear‐flattening, lip‐smacking, hind‐quarter touching, presenting, hip‐grasping, mounting, or penis diddling (genital touch) (Smuts and Watanabe 1990; Colmenares 1991a). The approaching individual was considered the initiator or “greeter”, and the other individual the recipient or “greetee” (Smuts and Watanabe 1990; Whitham and Maestripieri 2003; Dal Pesco and Fischer 2018). Definitions of “male greeting” vary across studies, with some requiring a minimum approach distance (e.g., 1 m) (Kalbitzer et al. 2015; Dal Pesco and Fischer 2018) while others do not (Smuts and Watanabe 1990; Whitham and Maestripieri 2003; Colmenares 1990). Some past studies have used relatively loose definitions of greeting, where approaches with lip‐smacking and/or ear‐flattening qualify (Colmenares 1990; Smuts and Watanabe 1990), while others have required a “gestural” component such as presenting or physical contact (Dal Pesco and Fischer 2018; Whitham and Maestripieri 2003), and others have been even stricter, requiring physical contact (Kalbitzer et al. 2015). Here, an inclusive definition was applied, with no minimum approach distance. Similar to work by Smuts and Watanabe (1990), I used ad libitum sampling, as I was not calculating hourly rates of greeting and instead was focused on the behavioral components of the greetings.

I coded all greetings between male adults, subadults, and medium to large juveniles, with at least one participant being an adult or subadult (*n* = 51). Interactions were only included where the sex of both individuals could be visually confirmed from the video footage or where identity, and therefore sex, was known. Birth years were unknown, so age classes were estimated using physical characteristics. Medium juveniles were defined as those who were larger than infants at the time of transitioning coat color but clearly smaller than the height of an adult female, large juveniles as those who had reached or were approaching the height of adult females but had not developed secondary sex characteristics, and subadult males as those who had increased testicular size, were of adult male height, but appeared lanky and less well muscled (Alberts and Altmann 1995; Altmann and Alberts 1987; Jolly and Phillips‐Conroy 2003). Adults were split into “old adults” and “young adults” (separate from subadults); individuals were considered “old adults” when they were visibly aged, with poor body condition and thinning/dull coats, while “young adults” appeared to be in their physical prime (Smuts and Watanabe 1990; Fraser and Plowman 2007). I fully coded all greeting events using an ethogram (available publicly: Muschinski and Carvalho 2023) developed based on those of Colmenares (1991b), Silk (2013), and Dal Pesco and Fischer (2018). Greeter and greetee identities were recorded when known. Age and sex class were recorded when individuals could not be identified. Of the 51 recorded interactions, 16 occurred between two known individuals, 13 had an unknown greeter and a known greetee, three a known greeter and unknown greetee, and 19 were between two unknown individuals (Table 1).

**TABLE 1:** Number of greetings observed between individuals.

Greetee identity											
Greeter identity	Aku*	Arrow*	Bulb*	Idoso**	Pao*	Patric***	Pequeno*	Quasimodo**	Unidentified adult male	Unidentified juvenile male	Unidentified subadult male
Aku*	0	0	1	0	0	0	1	0	0	0	0
Arrow*	1	0	0	0	0	0	0	0	0	0	0
Earl*	0	0	0	0	1	0	0	0	0	0	0
Pao*	0	1	0	0	0	0	0	0	0	0	0
Patric***	0	1	0	0	0	0	0	0	0	0	0
Pequeno*	1	0	0	0	0	0	0	0	1	0	0
Pez**	0	0	0	2	0	0	0	0	1	0	0
Quasimodo**	0	0	0	1	0	1	0	0	1	0	0
Redbeard*	0	1	0	0	4	0	0	0	0	0	0
Unidentified adult male	0	2	0	0	3	0	0	2	9	0	2
Unidentified juvenile male	0	1	0	1	0	0	2	1	3	0	0
Unidentified subadult male	0	0	0	1	0	0	0	0	2	1	2

*Note:* * young adult; ** old adult; *** large juvenile.

When the two minutes prior to the greeting were filmed, the context of each interaction was recorded (33/51 interactions). The context was classified as “neutral” where individuals were foraging, resting, grooming, or traveling and neither participant involved in aggressive interactions prior to the greeting (Smuts and Watanabe 1990). The context was labeled as “food present” where a high‐value, monopolisable resource was present within one meter of one of the individuals (e.g., one of the males sat along the edge of the firepit where refuse and food scraps are burnt) but the greeting was not preceded by active contesting of the resource. Greeting context was classified as involving “explicit conflict” where one of the two individuals had been involved in a chase or aggressive interaction in the two minutes prior to the greeting, or as “startled” where at least one of the individuals was vigilant towards a human or other potential threat (e.g., construction noise) prior to the greeting. This was recorded as the level of tension preceding the interaction is expected to affect the level of intensity of male greeting, that is, the social buffering hypothesis (Dal Pesco and Fischer 2018; Colmenares 1990). Coding was completed using Behavioral Observation Research Interactive Software (BORIS) version 7.10.2 (Friard and Gamba 2016). Data were cleaned using Python version 3.8.5 and R version 4.3.1. The data used in this study are available at http://doi.org/10.5281/zenodo.11097938.

### 2.3 Data Analysis

Signal use across greeter ages was calculated for ear‐flattening, lip‐smacking, presenting, embracing, mounting, penis diddling, hind‐quarter touching, and hip‐grasping. These calculations accounted for instances of poor visibility, and a table with sample sizes accounting for facial visibility can be found in the Supporting Information (Table S1). The total count of interactions for each age group can be seen in Table 2. Descriptive statistics of interactions involving juveniles are presented to provide limited insight into which behaviors are seen within the context of greeting earliest during development. Further analysis was not completed with interactions involving juveniles due to the small sample size (*n* = 11).

**TABLE 2:** Number of greetings observed for greeters and greetees of each age class.

Greeter age	Greetee age					Total
	Medium juvenile	Large juvenile	Subadult	Young adult	Old adult	
Medium juvenile	0	0	0	6	2	**8**
Large juvenile	0	0	0	1	0	**1**
Subadult	1	0	2	2	1	**6**
Young adult	0	0	2	26	2	**30**
Old adult	0	1	0	2	3	**6**
Total	**1**	**1**	**4**	**37**	**8**	**51**

For greetings involving only adults (*n* = 33) and for greetings involving adults and/or subadults (*n* = 40), I calculated the proportions of greetings that included physical contact and intense contact, were complete versus incomplete, and were reciprocal to compare to the existing baboon greeting literature. Intense physical contact was defined as genital touching, embracing, or mounting, in line with previous studies (Dal Pesco and Fischer 2018; Whitham and Maestripieri 2003). Approaches that involved facial signals but did not include presentation or physical contact from either individual were considered “incomplete,” in line with the terminology of Smuts and Watanabe (1990), while those that did were considered “complete.” I did not include a “partially complete” category, contrary to work by Smuts and Watanabe (1990), due to difficulty in defining the term.

Greetings were scored as “visually reciprocal” when the greeter and greetee both performed at least one behavioral element that was part of the greeting definition, which could include ear‐flattening or lip‐smacking (Colmenares 1991a). The proportion of visually reciprocal greetings was calculated in relation to all greeting events (i.e., complete and incomplete). Greetings were considered “physically reciprocal” where both individuals performed some type of physical motion, that is, either presenting or a contact behavior, following Dal Pesco and Fischer (2018). Physical reciprocity was calculated twice, once in relation to all greetings and once only in relation to complete greetings (i.e., where one of the two participants presented or initiated physical contact). The latter was to align with work by Dal Pesco and Fischer (2018), where interactions were only considered greetings if they included any of presenting, prancing, head bob, or a type of physical contact (i.e., a swaggering approach accompanied by ear‐flattening and lip‐smacking would not have been classified as a greeting, contrary to Smuts and Watanabe 1990). Bootstrapped 95% confidence intervals were calculated using the boot R package, version 1.3–28.1 (Davison and Hinkley 1997; Canty and Ripley 2022).

For each of these descriptive summaries, the datasets were screened based on visibility, to avoid biasing estimates (sample sizes presented in Table 5 alongside results). Contact and intense contact percentages were estimated from observations where either the face of both individuals was fully visible, or where at least one individual was seen to ear‐flatten or lip‐smack (e.g., 32 of 33 adult greetings met these requirements). This was to avoid including approaches in the estimate with no facial visibility, that were only classed as greetings due to the presence of contact behaviors that are more easily identifiable. This would have otherwise artificially inflated the estimated percentage of greetings that involved physical contact. Visual reciprocity was estimated twice, once only including observations where the full face was visible for both greeter and greetee (referred to as the strict estimate), and once where there was at minimum ear visibility for both individuals (the loose estimate). For example, in greetings by young adult males, 28/30 greetings had ear visibility, while 19/30 had full facial visibility. Percentages of greetings involving physical contact, intense contact, physical reciprocity, loose visual reciprocity, and strict visual reciprocity were calculated for (1) interactions including only adults and (2) interactions including adults and/or subadults (i.e., adult‐adult but also subadult‐subadult and subadult‐adult/adult‐subadult). Due to subadults only being involved in a total of 7 greetings (with either other subadults or adults), these were not analyzed as their own group, separately from the adult‐adult greetings.

#### 2.3.1 Context

Next, likelihood of physical contact was compared across contexts in greetings involving adults and/or subadults. Of the 40 greetings involving only adults and/or subadults, context could be recorded in 26 greetings. As only one observation was in a context involving explicit conflict, this was removed from analysis, reducing the sample size by one (remaining *n* = 25). Subadults and adult interactions were combined for this analysis due to the small number of observations. A logistic model including random effects for greeter/greetee identity was not possible due to the limited sample size and because it would have required the exclusion of any greetings involving unidentified individuals (13 of 24 greetings). A Fisher's exact test was used to compare likelihood of contact across contexts, as the expected minimum frequency was not five or more for all combinations. I hypothesized that likelihood of physical contact would differ across contexts and that it would be more frequent in more tense contexts (i.e., where a monopolisable resource is present) as a way to buffer tension (Dal Pesco and Fischer 2020; Hausfater and Takacs 1987; Colmenares 1990).

#### 2.3.2 Age Comparisons

##### 2.3.2.1 Age‐Class and Greeting Initiation

Studies in other baboon species have identified patterns in greeting role related to age and rank, possibly relating to greeting function. Fraser and Plowman (2007) found in hamadryas baboons that non‐prime males (referred to here as “old males”) initiated more greetings (referred to by Fraser and Plowman as “notifications”) than prime males (“young males”) and that these were more likely to be directed at prime males than non‐prime males. They suggested that one likely function of greeting behavior in hamadryas baboons is to signal submission. In olive baboons on the other hand, Smuts and Watanabe (1990) found that the dominant individual of a pair tended to act as the approacher. If greetings serve to signal submission or dominance in chacma baboons, we would expect that the likelihood to act as the greeter differs by rank. While rank data was unavailable for these individuals as they had not been individually identified at the time videos were recorded, male rank in COKY baboons typically peaks in young adulthood and then declines with age as physical condition and fighting ability decrease (Alberts et al. 2006; Beehner et al. 2006; Noë and Sluijter 1995; Packer 1979). Therefore, I aimed to explore the relationship between age‐class and greeting role. First, the relationship between age‐class and greeting role (greeter versus greetee) was explored using a Fisher's exact test, chosen because the expected frequencies assumption of a *χ* ^2^ test was not met. Second, a Fisher's exact test was again used to assess greetee age‐class preferences in greeters of given age‐classes.

##### 2.3.2.2 Age‐Class and Contact

Results from other species also suggest that contact behavior may differ across greetings depending upon the age classes of the greeter and greetee. In Guinea baboons, Whitham and Maestripieri (2003) found greetings involving lower ranking males were more likely to involve intense physical contact, particularly with the lower ranking male as the recipient. Among olive baboons, greetings between two old adults were more likely to qualify as “complete” compared to those involving other age combinations, whereas greetings between two young adults were more likely to stop before any gestural exchange occurred (Smuts and Watanabe 1990). Smuts and Watanabe (1990) suggested that this could be due to the results of increased jockeying for position and use of greetings to test relationships between young adults of similar physical condition and competitive power. Therefore, greetings between pairs of young adult males were expected to include contact behavior less frequently than those among other age classes (i.e., greetings between young adults and old adults, between old adults and old adults, between subadults and young adults, between subadults and old adults, or between subadults and subadults). To assess this, a *χ* ^2^ test with Yate's correction was used to compare the presence or absence of physical contact in greetings between pairs of young adults (*n* = 26 greetings) to those of other age combinations involving old adults, young adults, and subadults (*n* = 14 greetings). The lowest expected frequency was 4.55 and the minimum expected frequency assumption was therefore considered to have been met.

#### 2.3.3 Comparison Across *Papio*

To place results within the context of the *Papio* genus, a summary table was prepared (Table 8). Most published work on greeting in other baboons, with the exception of Guinea baboons, does not provide estimates and confidence intervals, making more in‐depth comparison difficult. Estimates for Guinea baboons presented in the table are from Dal Pesco and Fischer (2018). The estimate for percentage of greetings that are visually reciprocal for hamadryas baboons was calculated from Table 4 of Colmenares (1991a), which presents the hourly rate of asymmetrical greetings, symmetrical greetings, unreciprocated greetings, and total greetings for 11 time blocks. To calculate the proportion of unreciprocated greetings, the rates of unreciprocated greetings across the 11 time blocks were summed and divided by the sum of total greeting rates across the 11 time blocks. The proportion of reciprocated greetings was calculated as one minus the proportion of unreciprocated greetings. The definition for reciprocated greetings presented in Colmenares (1991a) aligns with the definition of visually reciprocal used here.

A minimum percent of all greeting attempts exhibiting contact and intense contact in olive baboons was estimated from Tables 2 and 3 in Smuts and Watanabe (1990). Only a minimum percentage could be calculated as percentages were only listed by behavior, with no overall category for contact. Table 2 in Smuts and Watanabe (1990) reported the number of greeting attempts, and median percentages of incomplete greetings, partial greetings, and complete greetings across age‐class combinations and for all greeting attempts. The total number of greeting attempts was 600, with 398 being recorded in Table 3 as partial or complete, allowing for the calculation of the percentage of incomplete greetings. Table 3 in Smuts and Watanabe (1990) reported the median percentages of partial and complete greetings that involved hip grasping, mounting, and genital touching. A minimum percentage of greetings including contact was calculated by determining the number of partial and complete greetings exhibiting mounting (48% of 398 observations) and dividing by the total number of greeting attempts reported in Table 2 (600). Mounting was selected as this was the physical contact category with the highest prevalence. This was also the method used to calculate the minimum percentage of greeting attempts classified as intense (exhibiting mounting, embracing, or genital touching). Where percentage estimates for certain species were not available (yellow baboon and hamadryas baboon), descriptors such as “present” or “often” are listed where appropriate in Table 8 in the results.

**TABLE 3:** Proportion of greetings where the greeter exhibited each signal of interest, split by age category.

Greeter age	Ear‐flattening	Lip‐smacking	Present	Embrace
Medium juvenile	0.25	0	1	0
Large juvenile	0	0	1	0
Subadult	0.5	0.5	0.17	0.33
Young adult	0.89	0.42	0.03	0
Old adult	1	0.75	0.33	0

Greeter age	Mount	Penis diddle	Hind‐quarter touch	Hip‐grasp
Medium juvenile	0	0	0	0
Large juvenile	0	0	0	0
Subadult	0.17	0.33	0.33	0
Young adult	0.03	0.07	0.13	0.03
Old adult	0.17	0	0	0

*Note:* Number of observations for each greeter age can be seen in Table 2. Further sample sizes based on facial visibility limitations can be found in Table S1.

## 3 Results

Data were collected for a total of 51 greetings, with 40 of these being between adults and/or subadults and 33 occuring between two adults. Ear‐flattening and lip‐smacking were the most common signals used by greeters and greetees in approaches between adults and/or subadults (Tables 3 and 4). Presenting by the greeter was seen in two of six approaches by old adult males, but only 0.03% (1/30) of approaches by young adults. Presenting by the greetee was seen rarely (in no greetings with old adult greetees, two of 37 with young adult greetees, and one in four with subadult greetees). Mounting was the type of physical contact most commonly exhibited by old male greeters (one of six greetings) and hind‐quarter touch the type of contact most common in approaches by young adults (four of 30 greetings). Mounting was not seen exhibited by any greetees, and hind‐quarter touches were present at a similar rate (14% and 12% respectively) in young adult and old adult greetees. Embraces were observed primarily among subadults, with two of six greetings by subadult greeters exhibiting an embrace and one of four greetings with a subadult greetee also involving embracing (Tables 3 and 4).

**TABLE 4:** Proportion of greetings where the greetee exhibited each signal of interest, split by age category.

Greetee age	Ear‐flattening	Lip‐smacking	Present	Embrace
Medium juvenile	0	0	1	0
Large juvenile	N.A.	N.A.	0	0
Subadult	1	0	0.25	0.25
Young adult	0.66	0.32	0.05	0.03
Old adult	0.62	0.57	0	0

Greetee age	Penis diddle	Hind‐quarter touch	Hip‐grasp	
Medium juvenile	0	0	0	
Large juvenile	0	0	0	
Subadult	0.25	0	0	
Young adult	0.08	0.14	0.08	
Old adult	0	0.12	0	

*Note:* Number of observations for each greeter age can be seen in Table 2. Further sample sizes based on facial visibility limitations can be found in Table S2.

Greetings by juvenile greeters most often involved presenting (eight of eight greetings), with little use of facial expressions (two of eight greetings). Of the nine greetings involving a medium juvenile, three involved physical contact. The two interactions involving a large juvenile, featuring the same large juvenile but with different adult male partners, involved physical contact. In addition to the signals reported in Table 3, grunting and grimacing, which have also been reported in other studies (Colmenares 1990; Hall 1962; Romero and Castellanos 2010), were seen in some greetings among adult/subadult males.

### 3.1 Completeness and Reciprocity

**FIGURE 1:**
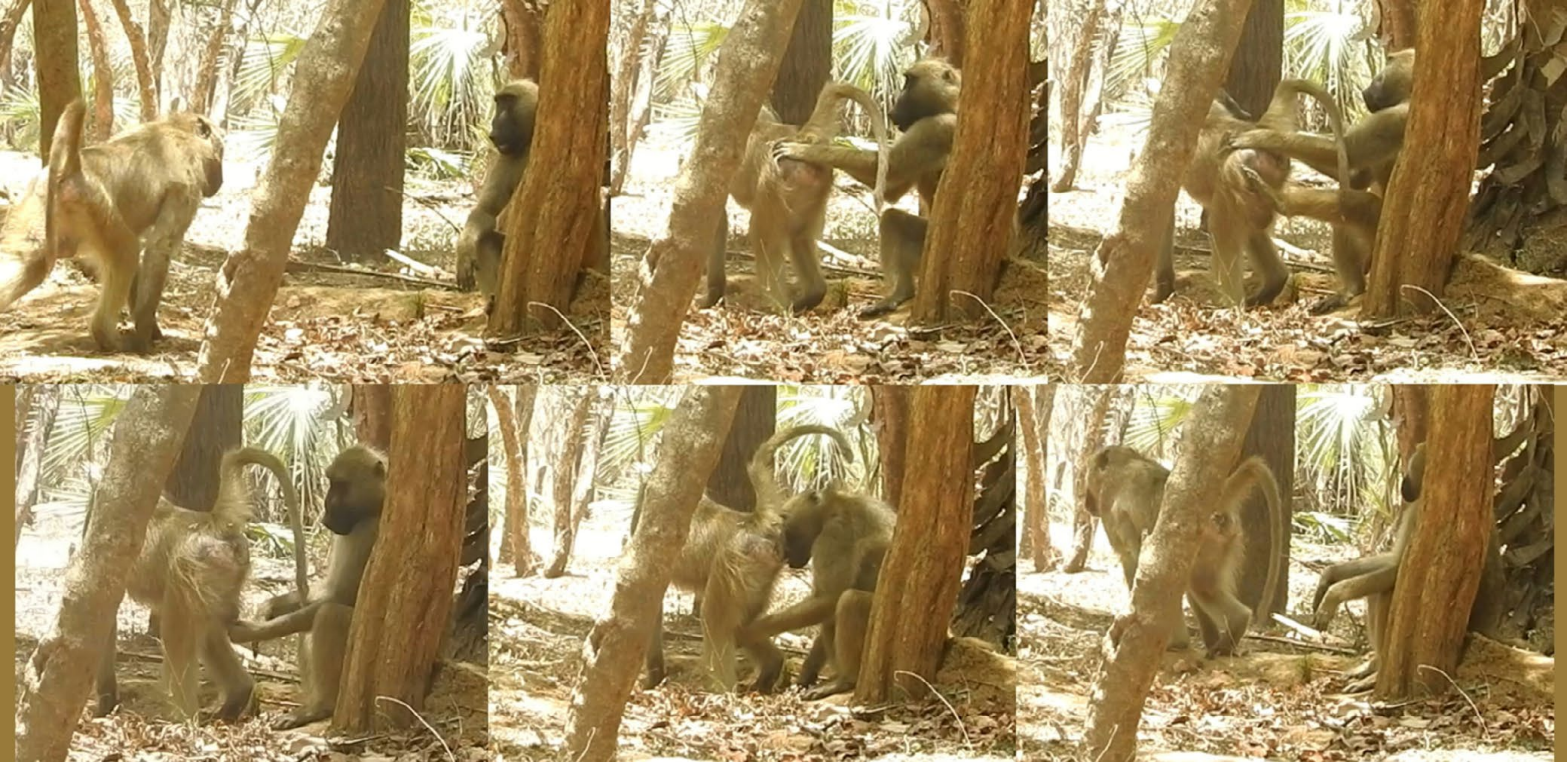
An example of the phases of an intense greeting observed between a young adult and an old adult male chacma baboon at Gorongosa National Park. From top left to bottom right: Approach with mutual gaze, lip‐smacking, and ear‐flattening; presentation and hip‐grasp; hip‐grasp extended with the addition of a hind foot; genital touching; genital inspection; distancing and ending of mutual observation.

Of all adult and/or subadult greetings, 65% were considered incomplete (i.e., involved only facial signals without further physical or tactile signals), which rose to 73% when considering greetings involving only adults. Thirty‐one percent of greetings between adults and/or subadults involved physical contact, with 13% involving intense physical contact (Table 5; see Figure 1 for an intense greeting example). When considering only greetings between adults (young or old), this was lower, with 22% of greetings involving contact and 6% intense contact (Table 6).

**TABLE 5:** Bootstrapped confidence intervals for the percentage of adult/subadult greetings which met the criteria for contact, intense contact, completeness, physical reciprocity, or general reciprocity.

Criteria	Mean	Lower bound	Upper bound	Sample size
Physical contact	31%	16%	45%	39
Intense contact	13%	2%	23%	39
Incomplete	65%	50%	80%	40
Physically reciprocal (out of complete greetings)	50%	24%	76%	14
Physically reciprocal (out of all greetings)	18%	6%	29%	40
Generally reciprocal (loose)	71%	56%	87%	35
Generally reciprocal (strict)	73%	54%	91%	22

*Note:* Sample sizes that each bootstrap is based on are provided and differ due to differences in visibility criteria (see methods). Physical reciprocity is presented as a percentage of greetings that involved presenting or physical contact by at least one participant. ‘Loose’ and ‘strict’ refer to inclusion of any greetings where at least ears were visible for both individuals or where the full face had to be visible for both individuals, respectively.

**TABLE 6:** Bootstrapped confidence intervals for the percentage of only adult greetings which meet the criteria for contact, intense contact, completeness, physical reciprocity, or general reciprocity.

Criteria	Mean	Lower bound	Upper bound	Sample size
Physical contact	22%	8%	36%	32
Intense contact	6%	0%	15%	32
Incomplete	73%	58%	88%	33
Physically reciprocal (out of complete greetings)	44%	12%	77%	9
Physically reciprocal (out of all greetings)	12%	1%	23%	33
Generally reciprocal (loose)	69%	52%	86%	29
Generally reciprocal (strict)	74%	54%	93%	19

*Note:* Sample sizes that each bootstrap is based on are provided and differ due to differences in visibility criteria (see methods). Physical reciprocity is presented as a percentage of greetings that involved presenting or physical contact by at least one participant. ‘Loose’ and ‘strict’ refer to inclusion of any greetings where at least ears were visible for both individuals or where the full face had to be visible for both individuals, respectively.

Approximately 70% of greetings (whether involving only adults or adults and subadults) could be considered visually reciprocal (i.e., both individuals performed at least one greeting signal). Physical reciprocity when calculated as a percentage of all greetings was 12% and 18% for greetings involving only adults and subadults, respectively. When calculated as a percentage of only complete greetings this was 44% and 50% (Tables 5 and 6).

### 3.2 Context

Of the 26 adult and/or subadult greetings where context was known, 17 occurred in neutral contexts, eight in close proximity to a monopolisable high‐value resource, and only one in a tense context following an aggressive interaction. Results of the Fisher's test indicated that there was no significant difference in contact between the two contexts included in analysis (neutral and resource‐present), *p* = 0.3942.

### 3.3 Greeter and Greetee Age

**FIGURE 2:**
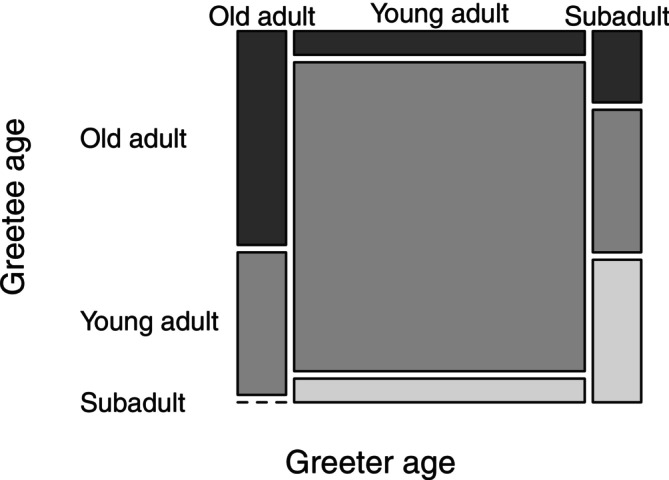
A mosaic plot showing the proportion of greetings by individuals from different age classes being directed towards old adults, young adults, and subadults.

A Fisher's exact test was used to assess whether there was a significant difference in role as greeter versus greetee between young, old adult males, and subadult males. No significant difference was found in role (*p* = 1). However, a significant difference was detected in the age‐class combination of greeter and greetee, *p* = 0.007. Greetings initiated by old adults were more likely to be directed towards other old adults compared to greetings by young adults (Figure 2). Similarly, young adults were more likely to initiate greetings towards other young adults, in comparison to old adult male greeters. Subadult greeters approached a combination of all age classes.

A *χ* ^2^ test could be conducted to assess whether contact behavior differed across greetings involving two young adults versus other age combinations (i.e., young adult–old adult, young adult–subadult, old adult–old adult, and subadult–subadult). The results indicated a significant association between age group and contact status, *χ* ^2^(1) = 4.36, *p* = 0.037. Specifically, greetings between young adults had fewer instances of contact than expected, while greetings in other age combinations had more instances of contact than expected. This appears to be driven by greetings between old adults and young adults and greetings involving subadults (Table 7).

**TABLE 7:** A contingency table showing the number of greetings for each greeter‐greetee age combination with and without physical contact.

Greeter–Greetee age combination	No contact	Contact
Old adult–Old adult	3	0
Old adult–Young adult	1	1
Young adult–Old adult	0	2
Young adult–Young adult	21	5
Young adult–Subadult	1	1
Subadult–Old adult	0	1
Subadult–Young adult	1	1
Subadult–Subadult	0	2

### 3.4 Chacma Baboons Within *Papio*

The bootstrapped estimates for contact, completion, and reciprocity are presented below (Table 8), along with those reported in and calculated from studies on other *Papio* species. Guinea baboons stand out among *Papio* as exceptional in terms of the proportion of greetings involving physical and intense contact. Chacma baboons are relatively similar in regard to the likelihood of physical contact and visual reciprocity to the other COKY baboons for which data are available but differ substantially in their percentage of incomplete greetings (Table 8). Compared to the 24% of greetings reported as incomplete in olive baboons, the percentage of incomplete greetings within this sample of chacma baboon greetings was noticeably higher at 65%.

**TABLE 8:** Comparison of male greetings across baboon species.

Species	Contact	Intense	Incomplete	Visually reciprocal	Physically reciprocal
Chacma (Gorongosa)	31% (including subadult greetings); 22% (excluding subadults)	13% (including subadults); 6% (excluding subadults)	65%	73%	50% of complete greetings (including subadults); 44% (excluding subadults)
Yellow^a^	Present	Present	N.A.	N.A.	N.A.
Kinda^b^	N.A.	N.A.	N.A.	N.A.	N.A.
Olive^c^	> 32%	> 32%	24%	Often	N.A.
Hamadryas^d^	Rare	Rare	N.A.	77%	N.A.
Guinea^e^	93.4%	59.2%	0.3%	N.A.	81.9%

^a^ Low rate of greeting observed—Hausfater and Takacs (1987).

^b^ No available data.

^c^ Smuts and Watanabe (1990).

^d^ Colmenares (1990), Colmenares (1991a), and Fraser and Plowman (2007).

^e^ Dal Pesco and Fischer (2018), Dal Pesco and Fischer (2020), and Whitham and Maestripieri (2003).

## 4 Discussion

It has been suggested that there is little to no male–male greeting in chacma baboons, with greetings that are far less elaborate than those of other COKY baboons (Kalbitzer et al. 2015), and physical greetings only seen when initiated by subadult natal males (Henzi et al. 2008), but this impression may stem partly from a lack of research on greeting in the species. Across only 65 h of footage, 33 adult (young or old, but excluding subadult) male greetings were observed, with 8 of these involving physical contact and two involving genital touching. While the rate of greeting per hour cannot be compared to those calculated in other studies, 31% of all adult and/or subadult greetings in this study were observed to involve contact, 13% intense contact, over 70% were visually reciprocal, and 50% of complete greetings were physically reciprocal. These align well with proportions seen in yellow, olive, and hamadryas baboons (see Table 8). The most noticeable difference between chacma baboons and these other three species was the percentage of greetings which were considered incomplete, that is, where only lip‐smacking and/or ear‐flattening occurred, with no presenting or contact behaviors. A markedly higher percentage of greetings were incomplete than in olive baboons and Guinea baboons (see Table 8). However, the term “incomplete” in itself is potentially misleading, as it suggests that such greetings are in some way interrupted or missing components, when they may simply be a simplified variant of greeting.

Dal Pesco and Fischer (2020) suggested that male–male greeting behavior in baboons follows a geographic cline in elaboration and ritualisation, with a large phylogenetic split between the southern (chacma, yellow, and kinda baboon) versus northern (olive, hamadryas, Guinea baboon) clades. Dal Pesco and Fischer point out that species where males are more spatially tolerant and affiliative also have the highest rates of greeting and the most intense greeting behavior, supporting suggested connections between human prosociality, larger social groups, and the evolution of ritual.

Previous research on greeting in chacma baboons is limited, with early studies by Saayman (1971) and Hall (1962) reporting limited instances of presenting and contact behavior between males. At odds with the remaining chacma literature, Saayman (1971) reported limited male–male coalitionary behavior, suggesting there may be within‐species variation in male cooperative behavior. This combination of cooperative behavior and contact greeting behavior is noteworthy given the rarity of both in other chacma baboon populations (Henzi and Barrett 2003) and how this aligns with the parallel gradients in physicality of greeting and male tolerance/cooperative behavior suggested by Dal Pesco and Fischer (2020). However, Henzi et al. (2008) argue that the coalitionary behavior described by Saayman (1971) differs from that described in yellow and olive baboons in that it involved primarily fear response signals, rather than the solicitations typically observed in other species. At the moment, data on coalitionary behavior among the chacma baboons in Gorongosa National Park is unavailable due to the recent identification of troop members and limited behavioral data available for the site so far, in part due to a reduction in data collection capacity during the COVID‐19 pandemic. However, pairs of young adult males were frequently observed by the author to sit within several meters of each other around a high‐value resource (i.e., firepit where food and refuse is burned), allowing each other to enter the pit to retrieve food items, while threatening or chasing off any other approaching males.

A lack of male coalitions is reported across a number of other chacma baboon populations (Henzi and Barrett 2003), including those residing in the Moremi Game Reserve, Botswana. Kalbitzer et al. (2015) studied approach behavior in chacma baboons residing in the Moremi Game Reserve, recording all approaches within 1 m. They recorded interactions as greetings only when non‐agonistic contact and non‐affiliative contact occurred (i.e., an approach in swaggering gait with lip‐smacking and ear‐flattening would not be considered a greeting, unlike in other greeting studies). They found that greetings occurred in about 7% of close proximity approaches (calculated from Supporting Information of Kalbitzer et al. 2015). Given the differences in definition and data collection, it is difficult to determine how the Gorongosa chacma baboons compare to those studied in the Moremi Game Reserve, though within‐species variation is not unlikely and would be a valuable direction for future research. Substantial behavioral variation can exist within species, reinforcing the need for additional in‐depth cross‐population studies with standardized methodology. Behavioral diversity in male greeting behavior within the same species, and even within the same population, has been reported in other species, for example mantled howler monkeys ( *Alouatta palliata* ) (Corewyn and Setchell 2019). Corewyn and Setchell (2019) found that male greeting patterns and frequency differed between groups and between dyads and suggested that individual group characteristics, ecology, and group history are likely to affect greeting behavior. Given the diversity of group demographics and ecosystems within (and across) *Papio* species (Fischer et al. 2019; Henzi and Barrett 2003), similar variation in male greeting behavior within baboon species can be expected.

Importantly, Gorongosa chacma baboon male greeting behavior appears to align broadly with that of the yellow, olive, and hamadryas baboons. Signals used are similar (see Table 3), with ear‐flattening, lip‐smacking, presenting, and various types of physical contact observed. It appears that overall, chacma greetings are more likely to involve only ear‐flattening and lip‐smacking, with no attempt at contact, compared to the other baboon species. However, contrary to Henzi et al.'s (2008) assertion that the full suite of greeting signals (i.e., including intense contact) is only observed in greetings between subadult natal males and adult males, this type of contact was also observed between pairs of adult males in the Gorongosa population. The prevalence of incomplete greetings and low rate of physical contact in chacma baboons supports the suggestion (Dal Pesco and Fischer 2020) that there is a gradient in greeting behavior within *Papio* which generally reflects levels of male–male tolerance and features of male–male relationships (e.g., coalition formation). Given the presence of similar types of male greetings in *Macaca* (De Marco et al. 2014; Riley et al. 2014; Silk 1994; Sugiyama 1971), having diverged from *Papio/Theropithecus* between 10.30 and 11.10 million years ago (Roos et al. 2019), a base level of ritualized greeting is likely ancestral and present across the genus (Dal Pesco and Fischer 2020), but with a divergence of function across the *Papio* genus. This study's sample size prevents further in‐depth comparison with other *Papio* species but does suggest that further research on greeting in southern clade baboons, and particularly in chacma baboons, is warranted. More between‐site variation in chacma baboon greeting behavior may exist than is currently documented in the literature.

Studying populations located in hybridisation zones may be particularly relevant to further understanding between‐ and within‐species differences in greeting behavior and their evolutionary drivers. Assessing whether differences in greeting behavior in hybrid populations parallels differences in coalitionary behavior and male tolerance would give insight into how strongly these characteristics are linked. Studies on greeting in captive populations of hamadryas and yellow baboon hybrids (Pelaez 1982) identified key differences in greeting form and movement, mismatches in greeting initiation and response, and areas of adaptation and behavioral flexibility in hybrid individuals. Studying these behaviors in wild hybrid populations would further our understanding of the evolutionary drivers shaping greeting and coalitionary behavior (Henzi et al. 2008).

### 4.1 Greeting Behavior Across Age Groups

While most of the greetings in the sample were between various classes of adults and/or subadults, the observed interactions did include nine greetings directed by medium or large juveniles towards adults, one greeting by a subadult towards a medium juvenile and one by an old adult towards a large juvenile. Unlike adult greeters, juvenile greeters were unlikely to use ear‐flattening and lip‐smacking, but all involved presentations (see Table 3). Lip‐smacking, while exhibited by baboons from an early age (Anthoney 1968; Chevalier‐Skolnikoff 1973), has been shown in cercopithecines to change throughout development as infants and juveniles mature, going through a development phase similar to human speech rhythm (Morrill et al. 2012). There are a wide variety of potential causes for the lack of lip‐smacking and ear‐flattening in juvenile‐led greetings, one being that use of ear‐flattening and lip‐smacking may change over time as their use of the signals develop. Ear‐flattening and lip‐smacking may also be less likely to occur in greetings by juveniles towards adults because these signals are often used to signal benign intent (Easley and Coelho 1991; Smuts 1985; Preuschoft 2000), which would not be needed given the low risk a juvenile greeter poses to an adult greetee. These minimal greetings by juveniles may also serve a different function than those observed in their adult counterparts and therefore consist of different behavioral components. Further research into the ontogeny of male greeting behavior would provide valuable insight into how use of these signals develop, how they differ from other interactions, and how rank and perception of risk influence signal use.

While formal comparison was not possible due to limited sample sizes, presenting appears more common by old male greeters (two of six greetings; presents performed by different old adults) compared with young adult greeters (one of 30 greetings). Old adult males also lip‐smacked in more of their interactions as both greeters and greetees than young adult males did (see Tables 3 and 4). These differences could be an indication of where we may expect to find differences due to rank; while rank was unknown for this study troop at the time videos were recorded, within savannah baboons old males past their physical prime are often lower ranking in comparison to young adult males (Alberts et al. 2006; Beehner et al. 2006). This aligns with findings by Colmenares (1990) that prime leader males in groups of hamadryas baboons were less likely to present in greetings, compared to older males. However, the limited sample sizes and the small number of individuals involved mean this could easily be a product of individual differences or sample size, rather than a consistent difference.

Across both young adults and old adults, ear‐flattening was more common than lip‐smacking for both greeters and greetees. As ear‐flattening and lip‐smacking are generally considered affiliative signals (Silk et al. 2018; Easley and Coelho 1991; Smuts 1985), it is possible that the combination of both may be used in particularly tense situations, but ear‐flattening may be the “default” in male greetings. Old adult males are often at a significant physical competitive disadvantage in comparison to younger males, meaning that they may particularly benefit from the clarity provided by the redundant use of ear‐flattening (seen in all five greetings by old adult greeters) together with lip‐smacking (seen in three of four greetings by old adult greeters with sufficient facial visibility) (Johnstone 1996). Alternatively, lip‐smacking may be an invitation for further progression of the greeting. Colmenares similarly noted that ear‐flattening could occur with or without other signals, often happening at a greater distance and that “within a short‐range, ear‐flattening is usually accompanied by lip‐smacking or grimacing depending on the level of fear of the displaying animal” (Colmenares 1990, 87).

When considering rates of contact, intense contact, completeness, and reciprocity in adult‐adult greetings compared to the sample of greetings including adults and subadults, we find that while rates of reciprocity are approximately the same across the two variations of the dataset, a higher proportion of adult‐only greetings appears to be incomplete (73% compared to 65%) and physical contact, particularly intense physical contact, is less likely (22% contact and 6% intense contact versus 31% contact and 13% intense contact). This may be because greetings involving subadults are lower risk as one of the greeting partners is not physically mature and tension in the interaction is therefore unlikely to be high. Subadults are also still residing in their natal troops, meaning that the other subadults/adults they are interacting with may be related to them. Finally, greetings by subadults in their natal troop may serve a different purpose than greetings between pairs of adults and could be exploratory or a way of eliciting support or affirming a bond. However, when comparing the data presented here for interactions involving only adults to those involving adults and/or subadults, it is important to note that the latter dataset also contains the former; further data collection focused on interactions involving subadults would give more insight into the strength (if any) of the differences identified here.

Analyses of the likelihood of greeting roles by age class and preferred recipient age class suggested that while no age class was more likely to be the greeter (as a proportion of total greetings involved in), different age classes did have preferred greetee age classes. Old adults appeared more likely to approach another old adult than a young adult, and young adults were similarly more likely to approach other young adults. Individuals in the same age class may be more likely to be close in rank, suggesting that greeting may be particularly relevant in situations where tensions may be higher. The comparison of contact behavior by age combination indicated that contact behavior is not independent of the age class combination of greeter and greetee. Greetings between pairs of young adults were significantly less likely than expected to exhibit contact behavior, while those in other age combinations involving young/old adults and subadults exhibited contact behavior more than expected. However, this seemed to be driven by young adult–old adult greetings and greetings involving subadults, as all three observed old adult–old adult greetings exhibited no physical contact. This is contrary to patterns that have been observed in olive baboons, where greetings between pairs of old adults appeared to be most reciprocal and balanced, with frequent physical contact (Smuts and Watanabe 1990). This difference may be linked to a difference in the function of greeting in olive baboons versus chacma baboons. Smuts and Watanabe (1990) suggested that the greetings between pairs of old adults often reflected their cooperative relationships, suggesting that their function may be at least partially related to maintaining and negotiating relationships, including cooperation. While it is currently unknown if this population of chacma baboons exhibits male coalitions, most chacma baboon populations do not (Henzi and Barrett 2003, Henzi and Barrett 2005; but see Saayman 1971), and as such the function of greeting in chacma baboons may instead be related to reaffirmation or negotiation of rank, which would align with the findings indicating higher rates of physical contact (a) in greetings involving subadults and (b) in greetings with a disparity in age class between greeter and greetee.

### 4.2 Consistent Definitions and Cross‐Site Comparisons

While developing criteria for the concepts of “completion,” “reciprocity,” and “greeting” itself for this study, it became evident that there is a lack of consistency across the baboon greeting literature that makes cross‐site and cross‐species comparison difficult. As demonstrated in Table 8, many of these characteristics have not been systematically collected across species. The field would benefit from standardization of definitions, especially as cross‐species comparison is critical for understanding the evolutionary drivers of greeting behavior in *Papio*. Open access publishing of produced datasets will also contribute greatly to cross‐site comparisons and meta‐analyses.

Studies also differ in their use of a distance cut‐off when recording greeting behavior. While many studies do not employ a distance cut‐off when defining greeting behavior (e.g., Whitham and Maestripieri 2003; Smuts and Watanabe 1990; Colmenares 1990) others do require the individuals to come within a certain distance of each other, for example, 1 m, for the greeting to be recorded (Kalbitzer et al. 2015; Dal Pesco and Fischer 2018). The use of a distance cut‐off may lead to the exclusion of greetings (or greeting “attempts” as they are referred to in some studies) which are discontinued by the greeter before the distance threshold required is crossed. However, due to differences in spatial tolerance across species, a “one size fits all” approach is unlikely to be successful when determining what an appropriate distance threshold may be. In‐depth assessment of the distance at which different signals are used may be helpful when determining such thresholds, giving insight into the distance at which a greeter's “intent to greet” may become more apparent to the recipient. In particularly tolerant species, the effect of using a distance threshold may be negligible because it is unlikely for greeting to occur without coming into close proximity.

Identifying greetings that are broken off early, after the initial swaggering gate accompanied by facial signals such as lip‐smacking and ear‐flattening, and collecting additional data on approaches which result in the distance threshold being crossed but without any recognized greeting signals would be valuable. Both of these sets of interactions, which fall outside of the stricter greeting definitions used in many studies, could provide insight into why certain greetings are aborted before entering close proximity, or why in some instances entering close proximity of another male does not require a greeting. Understanding both sides of this coin could aid in the study of greeting function. For example, if in a certain species the function of greeting behavior is to reduce tension and data are only gathered on approaches within 2 m with greeting behaviors displayed, we would miss out on information on those interactions where an approach with greeting was attempted but tension was too high and therefore aborted (i.e., 2 m threshold not met) and would also miss out on identifying pairs of individuals where close proximity was achieved but tension reduction was unnecessary and therefore no greeting behavior was displayed.

### 4.3 Limitations and Future Directions

While the results presented here help situate chacma baboon greeting behavior within the context of the *Papio* genus, there are several points on which direct comparison cannot be made to existing literature. The small sample size of male greetings means that the degree of uncertainty regarding estimates are higher, which should be considered when comparing to estimates from other species. Since not all individuals had been identified, bootstrapping could also not be weighted to account for overrepresentation of certain dyads. Furthermore, a per hour rate of greeting was not calculated, as several other studies have done, because the video data were not collected using randomized focal follows. The small sample size of this study in addition to lack of rank data allowed for only minimal testing of function. The lack of behavioral data from the field site makes it difficult to contextualize male–male greeting behavior in the study population within the larger behavioral repertoire (i.e., presence or absence of coalitionary behavior and levels of male tolerance).

## 5 Conclusions

Even with the limited sample size, the data presented here provide valuable insight into male greeting behavior in chacma baboons, which has a history of being poorly described and understudied. Estimates are provided regarding contact behaviors, reciprocity, and completion of greetings that allow for a basic comparison with the existing literature. Results demonstrate that male chacma baboons in Gorongosa exhibit the same behavioral greeting repertoire as described in other baboon species excluding the Guinea baboon, who have a more varied and ritualized repertoire. Chacma baboon greetings were found to have rates of contact and reciprocity broadly similar to greetings by yellow, olive, and hamadryas baboons, where reported. A far higher proportion of chacma greetings (65%) were found to be incomplete compared to other species, and intense physical contact (genital contact or mounting) between adults was rare (6%), particularly in comparison to Guinea baboons. Importantly, the presence of physical contact, including penis‐diddling, in adult greetings in the studied population indicates that this behavior is not restricted in chacma baboons to greetings initiated by subadults, as has previously been suggested. I furthermore highlight inconsistencies in previously used greeting definitions, suggesting areas for greater standardization to allow for easier cross‐species and cross‐site comparison.

## Author Contributions

**Jana Muschinski:** conceptualization (lead), data curation (lead), formal analysis (lead), funding acquisition (lead), investigation (lead), methodology (lead), project administration (lead), writing – original draft (lead), writing – review and editing (lead).

## Funding

This work was supported by the Department of Zoology, University of Oxford, Boise Trust Fund Grant. Clarendon Fund, Clarendon Scholarship. International Society for Human Ethology, Owen Aldis Scholarship. University of Oxford, St John's College, Special Grant.

## Supporting information


**Data S1:** Supporting Information.

## Data Availability

The data that support the findings of this study are openly available in Zenodo at http://doi.org/10.5281/zenodo.11097938.
